# Enhanced Electrocatalytic Oxygen Reduction Reaction of TiO_2_ Nanotubes by Combining Surface Oxygen Vacancy Engineering and Zr Doping

**DOI:** 10.3390/nano14040366

**Published:** 2024-02-16

**Authors:** Maged N. Shaddad, Prabhakarn Arunachalam, Mahmoud S. Hezam, Saba A. Aladeemy, Mamduh J. Aljaafreh, Sharif Abu Alrub, Abdullah M. Al-Mayouf

**Affiliations:** 1 Department of Chemistry, College of Science and Humanities in Al-Kharj, Prince Sattam Bin Abdulaziz University, P.O. Box 173, Al-Kharj 11942, Saudi Arabia; 2 Electrochemical Sciences Research Chair (ESRC), Chemistry Department, College of Science, King Saud University, Riyadh 11451, Saudi Arabia; 3 Physics Department, College of Science, Imam Mohammad Ibn Saud Islamic University (IMSIU), Riyadh 13318, Saudi Arabia

**Keywords:** TiO_2_ nanotubes, metal doping, oxygen reduction reaction, oxygen vacancy

## Abstract

This work examines the cooperative effect between Zr doping and oxygen vacancy engineering in anodized TiO_2_ nanotubes (TNTs) for enhanced oxygen reduction reactions (ORRs). Zr dopant and annealing conditions significantly affected the electrocatalytic characteristics of grown TNTs. Zr doping results in Zr^4+^ substituted for Ti^4+^ species, which indirectly creates oxygen vacancy donors that enhance charge transfer kinetics and reduce carrier recombination in TNT bulk. Moreover, oxygen vacancies promote the creation of unsaturated Ti^3+^(Zr^3+^) sites at the surface, which also boosts the ORR interfacial process. Annealing at reductive atmospheres (e.g., H_2_, vacuum) resulted in a larger increase in oxygen vacancies, which greatly enhanced the ORR activity. In comparison to bare TNTs, Zr doping and vacuum treatment (Zr:TNT–Vac) significantly improved the conductivity and activity of ORRs in alkaline media. The finding also provides selective hydrogen peroxide production by the electrochemical reduction of oxygen.

## 1. Introduction

In fuel cells, oxygen reduction reaction (ORR) is a prominent electrochemical reaction occurring in the cathodic compartment [1]. In acid solutions or alkaline media, there are two main pathways for oxygen reduction, namely direct four-electron reduction to H_2_O (in acidic media) or two-electron reduction to H_2_O_2_ [2]. An overpotential of 1.23 V (versus Reversible Hydrogen Electrode, RHE) [3] must be established to speed up the reaction. As far as fuel cell applications are concerned, platinum is the best material. However, platinum, in addition to suffering from low durability due to corrosion of the support (typically porous carbon), has a remarkably high cost, especially considering its extremely low utilization in the electrocatalysis process [4]. Many efforts have been made in recent years to resolve such Pt issues, for example, by supporting it onto conductive materials with high surface areas [5], or by alloying it with non-noble metals [6,7]. Other promising alternative methods [8] and nonprecious metals [9] are also being investigated. Alternatively, metal oxides based on non-noble metals can be used as a replacement for carbon support or to completely replace Pt, which greatly minimizes costs [10,11,12,13,14]. Metal oxides have, in fact, a few essential advantages. First, the metal in the metal oxide structure already exists in a high oxidation state (e.g., T^4+^ in TiO_2_), and is therefore more resistant to oxidation during fuel cell operation (which is the phenomenon causing carbon support degradation). Even when used as a support for Pt nanoparticles for example [15], there exists a strong Pt–metal oxide interaction that prevents the commonly observed problem of the aggregation and dissolution of Pt nanoparticles. Therefore, either as a support or as a standalone catalyst, metal oxides show exceptional stability when used as a cathode in fuel cells. In addition, metal oxides can be easily prepared by a variety of low-cost chemical methods, and a wide range of high-surface-area nanostructured thin films can be prepared by these methods with excellent producibility and scalability as well [16,17,18]. One of the most commonly used metal oxides is TiO_2_, and more interesting TiO_2_ nanotube (TNT) arrays, which have been extensively studied and used for a wide range of electrochemical applications [19,20,21]. Due to their particularly remarkable corrosion resistance in acidic and alkaline media [12], TNTs have been successfully examined for ORR catalysis [15,16,17,18]. TNTs are excellent electrode candidates as they offer both high surface areas, as well as direct electrical conduction pathways for charge carriers. In addition, TNTs make energetically favorable combinations with many material systems for different catalytic applications [22,23,24,25,26,27].

Oxygen reduction at the TiO_2_ surface is mainly a four-electron process [28]. It may, however, slightly depend on the fabrication procedure, TiO_2_ phase, and electrolyte properties. For example, on a single-crystal rutile surface, despite being largely a four-electron process as well, some oxygen species may go through a two-electron reduction to hydrogen peroxide [29]. In anodically prepared TiO_2_ electrodes, such as the ones in our study, it has been confirmed that a four-electron process is the dominant reduction process in alkaline media [17,30,31]. Two-electron reduction can, however, be enhanced by increasing the acidity of the electrolyte [30] by post-treatment of the surface [17], or by photo-induced reduction [31].

Researchers have recently discovered that self-assembled TNT arrays via anodization have unique nano-morphological and electronic properties [20,21,26]. The electrical conductivity of as-grown anodized TNT arrays is typically low, so their application in electro-catalysis and catalyst support is limited. In ORR catalysis, they typically serve as the support for noble metals or metallic alloys that acted as catalysts [15,16,32], mainly because TNTs alone are not very active in reduction reactions. Therefore, in order to use TNTs directly for the ORR process, metal or non-metal doping is often employed to produce oxygen vacancies in TNT structures and enhance their electrical features and reactivity [33,34,35]. However, fabricating highly efficient TNT electrodes with appropriate vacancy engineering processes is still a huge challenge.

Metal doping of TiO_2_ with Zr was reported to enhance different optoelectronic features. For example, the inclusion of Zr in TiO_2_ layers enhanced hybrid perovskite solar cell performance, which led to longer carrier lifetimes and greater charge carrier densities, perhaps due to fewer defects in bulk materials or at interfaces [36]. Previously, we investigated the cooperative effect of Zr ion doping and vacuum annealing of TNT arrays as an appropriate route to enhance the conductivity of TNTs [33]. It was shown that Ti^4+^ can be partially substituted by Zr^4+^, leading to a higher density of native defects, which reduces charge recombination and thus enhances the charge transfer process. Post-annealing under a reductive environment resulted in the creation of Ti^3+^ sites at the surface and oxygen vacancies in the bulk, respectively boosting the interfacial charge transfer and the conductivity of the TNTs [33]. It is thus interesting to explore if this dual Zr ion doping post-treatment combination would have a strong effect on ORR features.

In this work, highly conducting TNT arrays were obtained by anodization and subsequent heating at 450 °C in air (TNT–air), in nitrogen (TNT–N_2_), in hydrogen (TNT–H_2_), and in vacuum (TNT–Vac) atmospheres. The work also demonstrates the synergetic effects of Zr doping and annealing on TNT arrays under the same annealing environments. By using scanning electron microscopy (SEM) and cyclic voltammetry, we investigated how annealing gases can influence the surface chemistry of TNT array electrodes with and without Zr doping. The influence of preparation conditions on Zr:TNT electrode electrocatalytic performance is also studied.

## 2. Materials and Methods

### 2.1. Experimental Section

Ti foils (>99.5% purity, Alfa Aesar, thickness: 0.25 mm) were used to fabricate TNTs. Ammonium fluoride (NH_4_F) and zirconium (IV) acetylacetonate (C_20_H_28_O_8_Zr) were obtained from Sigma Aldrich. Ethylene glycol (EG) was acquired from BDH. All aqueous solutions were prepared with Milli-Q water (Merck Millipore, Darmstadt, Germany).

### 2.2. Fabrication of TNT Arrays

TNT arrays were produced using the electrochemical anodizing of Ti foil in two steps. TNTs were first cleaned ultrasonically and potentiostatically anodized for 30 min in a 2-electrode electrochemical cell with Pt foil as the counter electrode. Anodization was carried out at a constant voltage of 60 Volts using an electrolyte solution containing NH_4_F, water, and EG. Afterwards, the Ti foil was ultrasonically cleaned in millipore water for a few seconds for the next round of potentiostatic anodization, which lasted 3 h. After the anodized Ti foil was rinsed with water several times, it was annealed at 450 °C for 120 min at a ramp rate of 2 °C/min to obtain crystalline TNTs.

### 2.3. Fabrication of Zr:TNT Electrodes

Zr:TNT electrodes were fabricated by electrodepositing C_20_H_28_O_8_Zr in ethylene glycol (EG). In a three-electrode cell, the TNT was used as the working electrode, Pt as a counter electrode, and Ag/AgCl (3 M KCl) as a reference electrode, and electrodeposition was carried out. A potentiostatic cathodic deposition of Ag/AgCl was performed at −2.0 V with varying deposition times (2–15 s) with different Zr loadings (2 to 20 mC) in 1.0 M NaOH electrolyte. After that, the film was heated for 120 min in air at 450 °C (ramp rate = 2 °C/minute). Afterwards, both bare and Zr-doped TNT arrays were annealed a second time under vacuum, N_2_, or in air.

### 2.4. Characterization of Electrodes

In this study, XRD was measured using an X-ray diffractometer (Rigaku Miniflex 600, Tokyo, Japan) with a wavelength of 0.154 nm and a scanning rate of 3 per minute. The morphology of fabricated electrodes was investigated using Field-Emission Scanning Electron Microscopy (FE-SEM) (JEOL-7600F, Tokyo, Japan). Linear sweep voltammetry (LSV) was performed using an electrochemical workstation (Metrohm Autolab PGSTAT30, Herisau, Switzerland) with NOVA 1.8 software in a traditional three-electrode single-compartment Pyrex glass cell. In this experiment, saturated calomel (SCE) was used as the reference electrode and pure Pt foil was used as the auxiliary electrode.

## 3. Results and Discussion

### 3.1. Crystalline Properties of Zr:TNT Arrays

**Figure 1:**
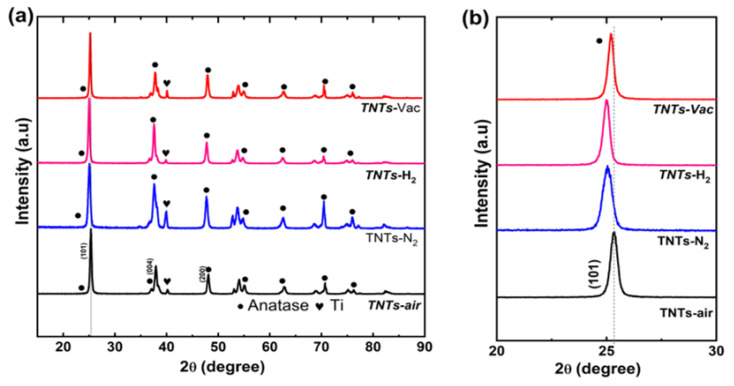
(**a**) X-ray diffraction patterns of bare TNTs (black), TNT–N_2_ electrodes (blue), TNT–H_2_ electrodes (magenta) and TNT–Vac (red) electrodes obtained through annealing under atmosphere (**b**) and enlarged view of fabricated electrodes.

Ti foil is anodized to make the TNTs. In fact, the as-grown TNTs are amorphous [37,38]. The post-annealing treatment is important after that to improve their structural, morphological, and electrical properties [37,39,40]. Figure 1a shows the XRD patterns for the TNT samples post-annealed under air, N_2_, H_2_, and vacuum. Upon analyzing the XRD, the crystalline nature of all samples showed a pure anatase phase (JCPDS card no. 21-1272) without displaying any secondary phases (except the peaks of the Ti metal from the substrate), despite being heat-treated in four different atmospheres (air, N_2_, H_2_, and vacuum). In agreement with previous results, the (101) anatase peak at ~25.3° dominated the other peaks in the diffraction patterns [37,39], and this was the case for all annealing environments. A magnified image of the (101) region is shown in Figure 1b. By studying the (101) anatase peak, it can be noticed that different gas environments bring about slightly different peak positions. This can be attributed to the fact that different annealing environments result in different levels of lattice strain in the samples [41,42]. Compared to the TNT–air sample, all other environments resulted in reduced strain, which can be observed from their smaller peak positions. The shifts in peak positions were, however, not very significant, with the largest shift of about 0.4°under H_2_ annealing.

### 3.2. Morphological Features of Zr:TNT Arrays

**Figure 2:**
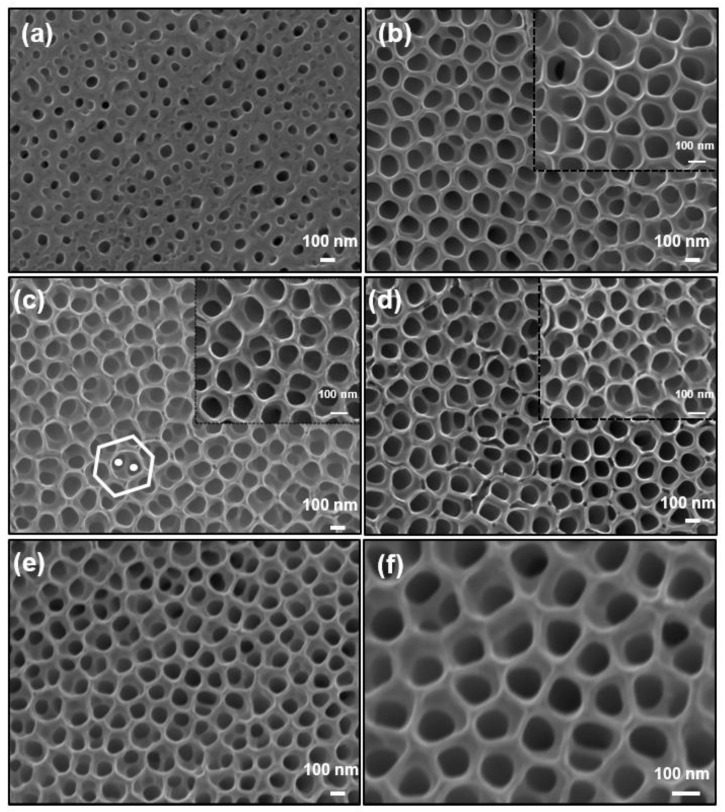
FE-SEM top-view images of as-prepared TNTs (**a**), TNTs annealed in air (**b**), TNTs annealed in N_2_ (**c**), TNTs annealed in H_2_ (**d**), TNTs annealed under vacuum (**e**,**f**).

SEM images of non-annealed TNTs and TNTs annealed in different annealing conditions are shown in Figure 2. In all samples, the adoption of the two-step anodization process resulted in highly ordered structures. In two-step anodization, TNTs are grown in the first anodization step of the Ti foil (which usually has a corrugated irregular surface) at a high voltage of 60 V, at which the tube dissolution is significant. This results in a TNT disordered array that is easy to be removed. Cleaning steps after the first anodization step remove the remnants and “delineate” a nanoconcave pattern on the Ti sheet. The first anodization also removes, to a large extent, the native oxide layer on the Ti foil. The delineated Ti substrate is then used in the second anodization step at a lower voltage to form a highly ordered uniform array of TiO_2_ nanotubes [43,44,45,46]. Our grown TNTs were smooth and well-aligned, and no apparent defects were noticed. The as-prepared sample has a top amorphous layer, and the nanotubes are grown underneath [43]. This layer does not appear on the surface of annealed samples. Considering the grown TNT pattern, each nanotube is generally surrounded by six other nanotubes resulting in a hexagonal closed packed pattern, in agreement with previous work [45,46]. However, it can be noticed that we have two levels of nanotube growth: upper hexagonal nanotube pores on the top (highlighted by a hexagon in Figure 2c) and two lower-level inside pores (highlighted by circles in Figure 2c) that have smaller diameters, making a double-layer lotus-root-shaped nanostructure. This structure has been reported to have low values of the second anodization voltage, and more than two inside pores can be observed [46]. In our growth conditions, we had 1–2 inside pores, the size of which were substantial compared to the size of the upper pore, which resulted in deviations from the hexagonal close-packed structure at many locations. In general, thermal treatment under air, N_2_, H_2_, and vacuum does not alter the morphological features of TNTs either on their surface or on the inside. The characteristic parameters of the different nanotubes were composed of NTs 147 ± 5 nm in diameter with a length of 900 ± 50 nm. Based on the image analysis, the surface density was estimated to be ~150 tubes/square meter. The TNTs maintained their structural integrity after annealing. The nanotubes appeared to be of high quality and can be used for electrocatalytic applications.

### 3.3. Electrochemical Performance of TNT Arrays

#### 3.3.1. Electrochemical Performance of TNT Arrays without Zr Doping

**Figure 3:**
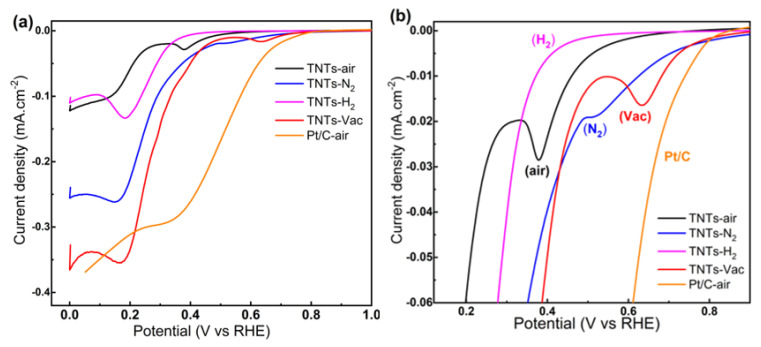
(**a**) LSV for a TNT electrode at a scan rate of 10 mVs^−1^ immersed in 1.0 M NaOH and annealed under air, N_2_, H_2_, and vacuum conditions; (**b**) enlarged view of the figures.

Linear sweep voltammetry was first used to characterize pure TNTs (without the Zr deposition step) thermally treated under different atmospheric conditions. Using 1.0 M NaOH at 10 mV/s, TNT, TNT–N_2_, TNT–air, and TNT–Vac array electrodes were investigated for ORRs measured between 1.0 and 0 V versus RHE. The results are shown in Figure 3a, with Figure 3b showing the measurements. As a benchmark electrode material, a commercial Pt/C catalyst was also tested and is presented in Figure 3. In the TNT–air electrode, two distinct reduction peaks are seen at 0.38 V and 0.14 V vs. RHE, with cathodic peaks indicating mass transport-controlled reduction and peak currents of 0.11 mA cm^−2^. All electrodes studied under different annealing atmospheres showed distinct cathodic peaks, but TNT electrodes processed under vacuum performed the best electrochemically. Remarkably, the TNT–Vac electrode showed much greater ORR onset potential, as well as a superior current density of about 0.35 mA cm^−2^, which is considerably greater than the TNT–air sample (0.09 mA cm^−2^), and is also comparable to that of the commercial Pt/C electrode. On the other hand, the LSV voltammogram of TNT–Vac indicates that, at the measured potentials, cathodic currents are lower than those of the Pt/C materials. In comparison with those for TNT–N_2_, TNT–H_2_, and TNT–air electrodes, TNT–Vac’s oxygen reduction potentials shifted to a more positive position, indicating a higher catalytic activity. This indicates that TNT–Vac is more efficient in reducing oxygen than TNT–N_2_, TNT–H_2_, and TNT–air electrodes. Moreover, the shifted potentials of TNT–Vac suggest improved catalytic activity towards oxygen reduction.

#### 3.3.2. Electrochemical Performances of Zr-Doped TNT Arrays

For Zr-doped samples, the Zr loading was first optimized for the best catalytic activity. Different Zr loadings were tested on TNTs during the cathodic electrodeposition process, and the optimal Zr content was determined after testing different applied charges. In Figure S1, LSV plots are shown for a TNT electrode with different Zr loadings (2 to 20 mC) in 1.0 M NaOH electrolyte. The results show that the optimal electrodeposited charge is 2 mC. Thus, unless otherwise specified, the optimized Zr-doped TNT electrodes are referred to as Zr:TNTs throughout the manuscript.

**Figure 4:**
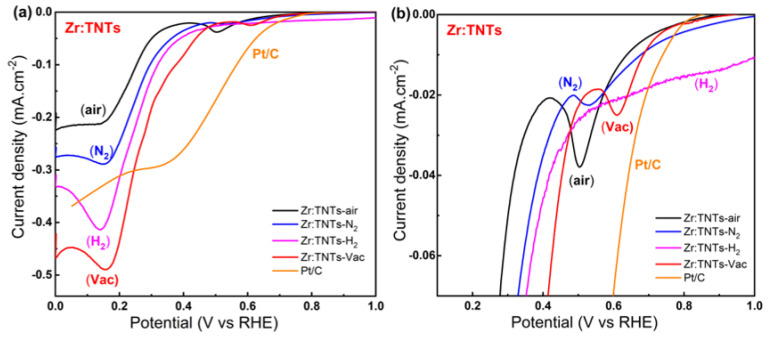
(**a**) LSV for a Zr:TNT electrodes at a scan rate of 10 mVs^−1^ immersed in 1.0 M NaOH and annealed under air, N_2_, H_2_, and vacuum conditions; (**b**) enlarged view of the figures.

The optimized Zr-doped TNT electrodes were then used to investigate the combined effect of Zr incorporation (conditions) and post-annealing under different environments on ORR activity in alkaline media. The results are shown in Figure 4. As shown in Figure 4a, the LSV plots show the effect of annealing atmospheres in 1.0 M NaOH on Zr:TNT electrodes. A magnified view of the plots is shown in Figure 4b. It can be noticed that chemically irreversible reduction peaks occur at potentials of 0.61 V and 0.16 V vs. RHE in Zr:TNT–Vac. It can be clearly observed that Zr:TNT–Vac has also the highest current density (now even higher than the commercial Pt/C electrode) and ORR onset potential as well. The electrochemical results indicated that both Zr doping and vacuum annealing on TNT electrodes increased ORR activity more than N_2_-annealed, H_2_-annealed, and air-annealed Zr:TNT substrates. The electrochemical tests also confirm the importance of the vacuum annealing effect on Zr-doped TNTs. Overall, the Zr doping and vacuum annealing of TNTs proved to be a successful combination for increasing ORR activity, in agreement with the reported literature on other electrochemical systems [33,47,48].

#### 3.3.3. Effect of Oxygen Concentration on the Reduction Peaks in Vacuum-Annealed Zr-Doped TNT Arrays

**Figure 5:**
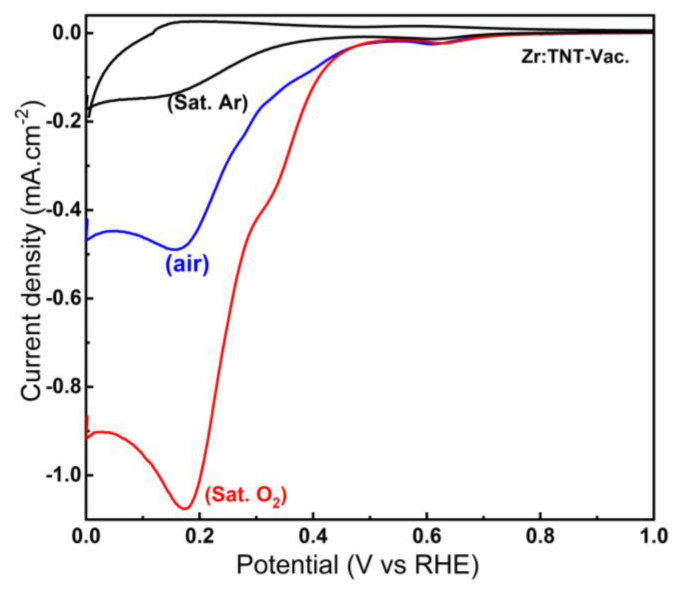
CV for a Zr:TNT–Vac electrode at a scan rate of 10 mVs^−1^ immersed in 1.0 M NaOH purged for 20 min with pure argon, air, and pure oxygen.

The origin of the two cathodic peaks, and if they are directly related to oxygen reduction at the surface of the champion Zr:TNT–Vac sample, can be investigated by changing the gas purging environment during LSV measurements. Using alkaline solutions pumped with pure O_2_, air (~20% O_2_), and pure argon under vacuum, the effect of oxygen concentration could therefore be investigated for the Zr:TNT–Vac electrode. LSV diagrams for the different oxygen concentrations in 1.0 M NaOH are shown in Figure 5. Under vacuum, LSV results for the electrode show only a small reduction peak at 0.13 V after completely deoxygenating the solution. Both reduction peaks started to be observed after purging the solution with air. By purging with pure oxygen gas, the current increases significantly with increasing oxygen concentrations. The results of this experiment prove that these two peaks are the result of oxygen reduction at the Zr:TNT–Vac array surface, and we speculate that the reduction process involves two sequential reactions. At the first peak at potential 0.63 V, oxygen is reduced by two electrons to give H_2_O_2_ (or more correctly HO^2−^), which is followed by the second peak at potential 0.18 V vs. RHE by a further two electron reductions to give water. We previously observed a similar two-step reduction mechanism for N_2_-annealed TNTs [17]. Based on the above results, the Zr:TNT–Vac electrode materials exhibit excellent ORR catalytic activity. Lastly, these results show that these electrode materials have potential applications in the field of ORR.

**Figure 6:**
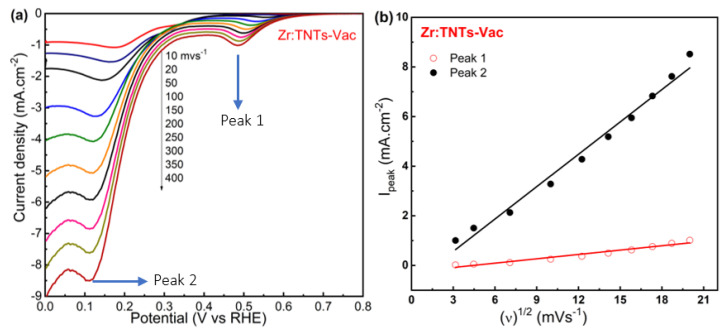
(**a**) LSV at different scan rates for a Zr:TNT–Vac electrode in 1.0 M oxygen-saturated NaOH; (**b**) plot for peak currents versus square root of scan rate.

We further investigated the effect of scan rate on the Zr:TNT–Vac sample under a saturated O_2_ measurement environment. Figure 6a shows LSV diagrams of the Zr:TNT–Vac array samples at different scan rates in 1.0 M NaOH solution that was O_2_-saturated. Figure 6b shows the relationship between the two cathodic peak currents and the square root of the scan rate. For the Zr:TNT–Vac electrode, both oxygen reduction peak currents increased linearly with potential scan rate squared. This indicates that oxygen reduction is diffusion-controlled. Moreover, the diffusion rate of oxygen into the electrode increases with the potential scan rate. Based on this, it is likely that the diffusion of oxygen from the solution to oxygen vacancies in the electrode is controlling the overall ORR process. Therefore, it can be concluded that oxygen reduction is diffusion-controlled in Zr:TNT–Vac electrodes.

#### 3.3.4. EIS Measurements

**Figure 7:**
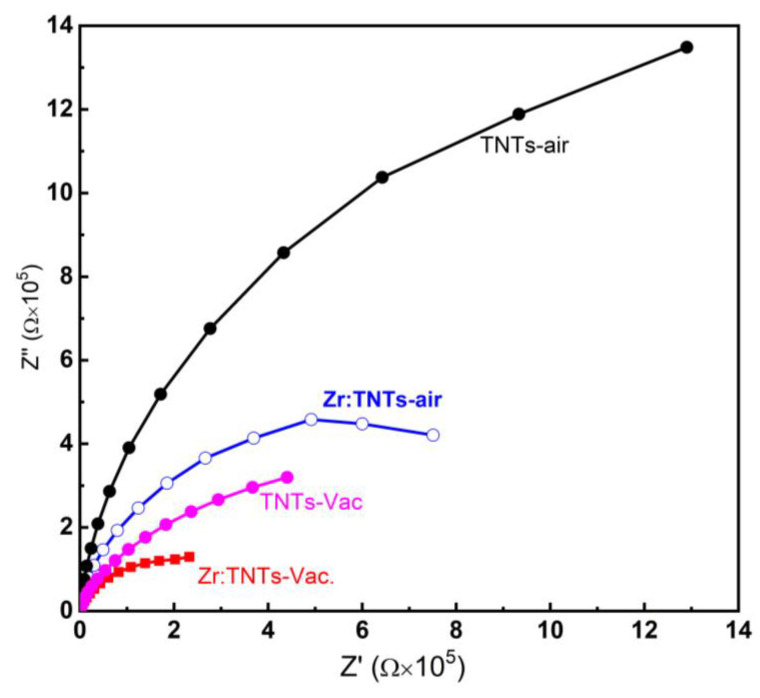
Nyquist plots of fabricated TNT array films, TNT–Vac, Zr:TNT–air, and Zr:TNT–Vac substrates.

EIS measurements were performed on TNT films before and after Zr doping annealed under air and vacuum environments in order to determine the electrode reaction kinetics and charge-transfer resistance. Figure 7 shows typical Nyquist impedance plots (Z’ versus Z”) of the EIS data acquired for the TNT–air, TNT–Vac, Zr:TNT, and Zr:TNT–Vac electrodes obtained in the frequency varying between 10^−2^ and 200 kHz. Zr doping alone (blue arc) resulted in a reduced arc radius compared to that of the undoped sample (black arc), indicating that Zr doping resulted in a higher conductivity of the electrode. Zr doping also enhanced conductivity for the vacuum-annealed samples (magenta and red arcs). Similarly, vacuum annealing resulted in reducing the charge-transfer resistance as well for pure and Zr-doped electrodes. Therefore, the Nyquist plot for the Zr:TNT–Vac electrode has the smallest arc radius, indicating the remarkable synergetic effect of Zr doping and vacuum annealing. This combined effect clearly resulted in a smaller charge-transfer resistance, which again demonstrates superior electrochemical performance. The enhanced charge transfer kinetics of the Zr:TNT–Vac sample will manifest as an efficient ORR catalysis. Overall, the introduction of Zr doping and vacuum annealing to the TNT–air electrodes resulted in improved electrochemical performance, thus making them a promising candidate for oxygen reduction reactions.

#### 3.3.5. Mechanisms of ORR Enhancement

The ORR process takes place at the TNT surface, and is therefore an atomistic process in nature. The effect of annealing under a reductive atmosphere (e.g., vacuum) on the TNT surface can be explained as follows. The reductive environment results in a higher density of oxygen vacancies, which are very diffusive to the surface [49]. At the oxygen vacancy sites on the surface, unsaturated Ti^3+^ (instead of saturated Ti^4+^) sites will be exposed to oxygen molecules and are thus ready to work as oxygen reduction sites. At the same time, oxygen vacancies are native defects that serve as electron donors, and increasing their density will therefore be reflected in a higher conductivity of the nanotubes, confirming the EIS results. The higher conductivity will improve electron transfer through the TNT bulk (by decreasing bulk carrier recombination events) until reaching the external circuit and the electric load of the fuel cell are reached, thus ultimately increasing the charge collection efficiency of the electrode. In a nutshell, oxygen vacancies created by the reductive environment improve photocatalytic activity in two ways: the creation of exposed Ti^3+^ ORR sites at the surface, and increasing free electron density and thus conductivity in the bulk of nanotubes.

**Figure 8:**
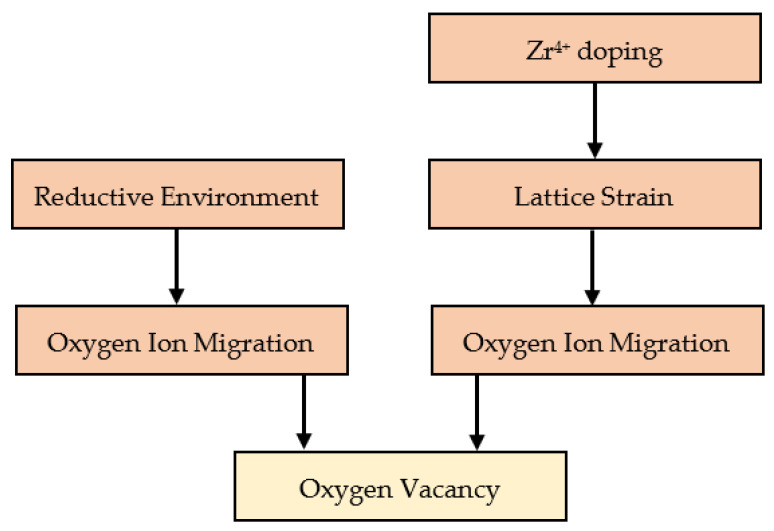
A schematic showing the different possible paths to generate oxygen vacancies in TNTs.

The effect of Zr doping is more difficult to explain. It has to be mentioned first that Zr deposition was performed after TNT growth and not during the growth of the TNTs. Post-annealing after that will cause Zr atoms to diffuse into the bulk. However, a large concentration of Zr atoms is expected to be close to the surface. Although the formation of ZrO_2_ nanoparticles or nanoclusters is a possibility that cannot be ignored [50], we previously showed by X-Ray Photoelectron Spectroscopy (XPS) that Zr^4+^ ions replace Ti^4+^ ions in the lattice [33]. Therefore, both Zr^4+^ and Ti^4+^ have the same oxidation state and so doping with Zr is not expected to directly increase the free carrier density in TNTs. In addition, metal doping generally increases the formation energy and diffusion coefficient of oxygen vacancies in the lattice [51]. Both scenarios are against the observed higher conductivity observed in EIS measurements, and the higher photocatalytic activity as well. In fact, the enhanced conductivity can be explained as follows. In the TiO_2_ anatase lattice, Ti^4+^ lies at the center of TiO_6_ octrahedra, making a coordination number of 6 for the Ti^4+^ ion. For such a combination, the ionic radius of Ti^4+^ is 60.5 pm, while it is 72 pm for Zr^4+^ in the same lattice [52,53]. This slightly higher ionic radius of Zr^4+^ will induce a strain in the lattice. To alleviate this strain, nearby oxygen ions can escape to the surface, leaving oxygen vacancy donors that will thus increase the density of free electrons in the TNTs [54]. This process will be energy-supplied by heat in the annealing step. If annealing is further performed in a reductive environment, the formation of oxygen vacancies that will readily take place, as well as the creation of Ti^3+^(Zr^3+^) sites on the surface, will amplify the effect initiated by the presence of Zr^4+^ dopants, thus explaining the combined effect of both Zr doping and vacuum annealing. In summary, oxygen vacancies can be created directly by the reductive environment and indirectly by Zr doping, which result in a higher density of free carriers in the bulk and unsaturated Ti^3+^(Zr^3+^) sites at the surface. Figure 8 shows a schematic of the two paths available to generate oxygen vacancies in our samples.

**Figure 9:**
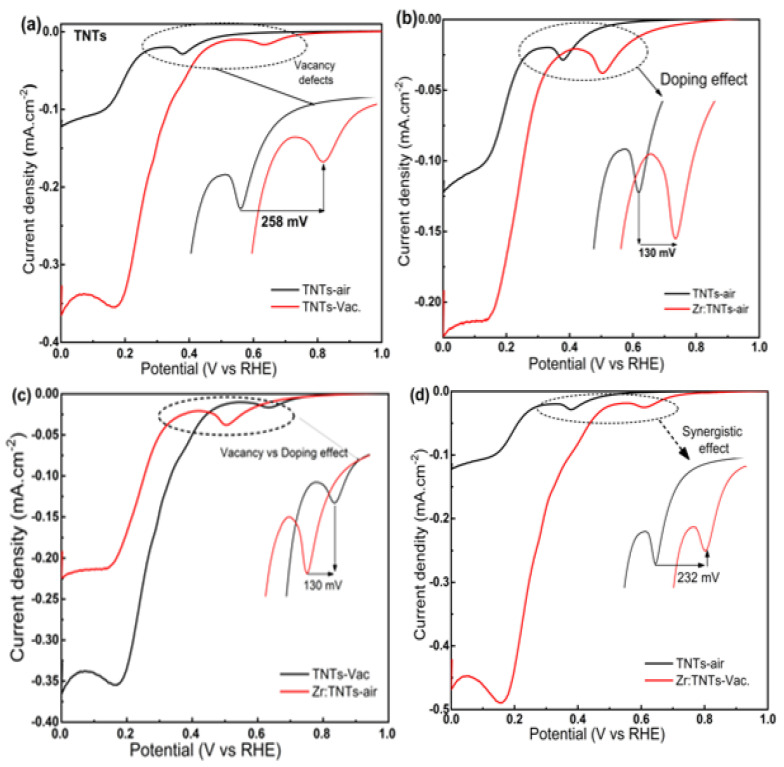
LSV diagrams for (**a**) TNTs–air vs. TNT–vacuum electrodes to show the effect of oxygen vacancies on bare TNT electrodes (**b**) TNTs–air vs. Zr:TNT–air electrodes to show the effect of Zr doping, (**c**) TNTs–Vac vs. Zr:TNT–air electrodes, and (**d**) TNTs–air vs. Zr:TNT–Vac electrodes to show the synergistic effect for both kinds of defects (vacancy and doping). All results were measured at a scan rate of 10 mVs^−1^ immersed in 1.0 M NaOH.

This work and our previous studies [33,47,48] confirm the fact that the vacuum annealing process is more responsible for enhanced electrochemical activity than Zr doping. In Figure 9, different LSV diagrams from Figure 4 and Figure 5 are presented for different comparisons that can shed light on the weight of each of the two parameters. Figure 9a illustrates the vacancy effect on TNT substrate. In comparison to TNT–air, vacuum annealing significantly improved TNT conductivity and reactivity, with a 258 mV positive potential shift for oxygen reduction. As illustrated in Figure 9b, the effect of doping on TNT substrates is also shown. As a result of Zr doping over TNTs, an oxygen reduction shift of 258 mV was observed. Figure 9c shows the comparative effect of vacancies and doping. Figure 9d demonstrates the synergetic effect of Zr doping and vacuum annealing on TNTs. Compared with TNT–air, the synergetic effect of Zr doping and vacuum annealing showed a 232 mV positive potential shift. Therefore, vacuum annealing reduces TNT potential more effectively than Zr doping. This indicates that Zr doping improves oxygen reduction performance when vacuum annealing is present. This suggests that vacuum annealing is a key factor in TNT oxygen reduction.

#### 3.3.6. ORR Catalytic Stability

**Figure 10:**
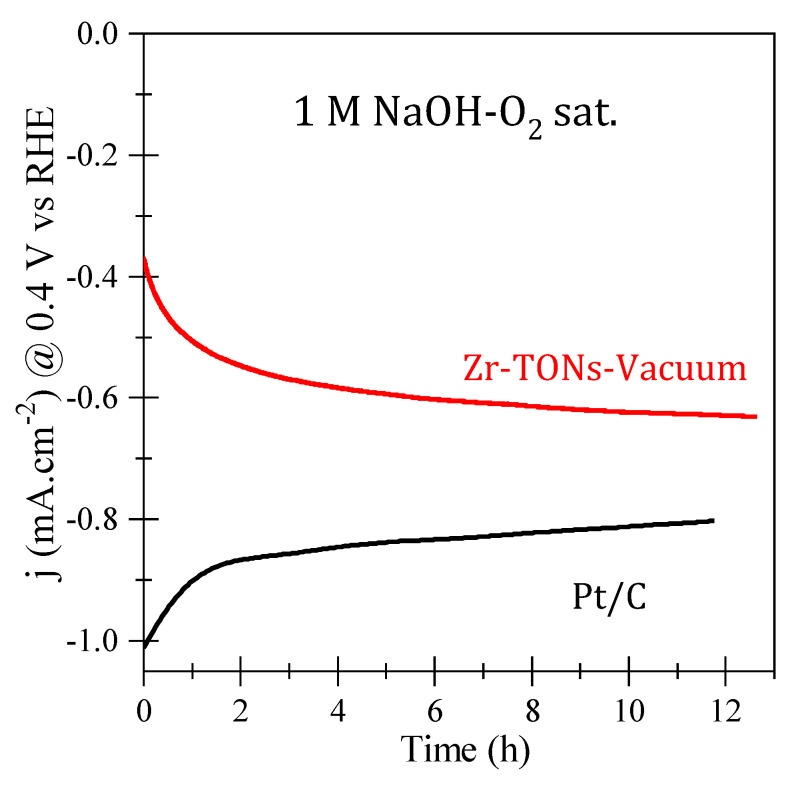
ORR stability chronoamperometric curves for Zr–doped vacuum-annealed TNTs, as well as Pt/C electrodes in 1.0 M oxygen-saturated NaOH electrolyte.

ORR catalytic stability was investigated for the optimal Zr-doped vacuum-annealed electrode, and the chronoamperometric (I−t) curves under a saturated oxygen electrolyte environment for both the optimal TNT and Pt/C electrode are shown in Figure 10. The Pt/C electrode deteriorates quickly by ~20% over 12 h. On the other hand, the current in the optimal TNT electrode increases by more than 50% before reaching a saturation current density of −0.62 mA/cm^2^ at 0.4 V vs. RHE, indicating the long-term stability of the electrode. Similar stability behavior has been reported in the literature for metal oxide electrodes in general and for the TNT morphology as well [12,15].

## 4. Conclusions

Overall, we demonstrate that Zr doping and vacuum annealing have synergistic effects on improving TNT surface activity, which results in improved ORR performance in alkaline media. Vacuum annealing results in donor-type oxygen vacancies, which increase the conductivity of the TNT bulk and also create unsaturated Ti^3+^(Zr^3+^) sites at the surface that work as active oxygen reduction sites. Zr doping can also result in the creation of oxygen vacancies that will relax the lattice when Ti^4+^ ions are replaced by Zr^4+^. The synergetic effect resulted in a remarkable improvement in ORR activity and stability for the optimized electrode. Compared to commercial Pt/carbon electrodes, higher current density was obtained, however at the cost of the appearance of two-electron reduction peaks, making the process slower compared to the complete four-electron process with Pt. On the other hand, these TNTs can be efficiently applied for the production of hydrogen peroxide by selective ORR.

## Data Availability

Data are contained within the article and supplementary materials.

## Supplementary Materials

The following supporting information can be downloaded at: https://www.mdpi.com/article/10.3390/nano14040366/s1, Figure S1: LSV diagrams for TNT electrodes with different ZrO_2_ loading (2 to 20 mC) at a scan rate 10 mVs^−1^ immersed in 1.0 M NaOH and annealed under air.
