# Supplementary material for: Enhanced Electrocatalytic Oxygen Reduction Reaction of TiO2 Nanotubes by Combining Surface Oxygen Vacancy Engineering and Zr Doping
Source: Nanomaterials (Basel). 2024 Feb 16;14(4):366. doi: 10.3390/nano14040366 (PMC10892297; doi:10.3390/nano14040366)
Supplement: Supplementary file 1 [file nanomaterials-14-00366-s001.zip › nanomaterials-2800266-supplementary.pdf]

Supplementary Materials

Enhanced Electrocatalytic Oxygen Reduction Reaction of TiO2 Nanotubes by Combining Surface Oxygen Vacancy Engineering and Zr Doping

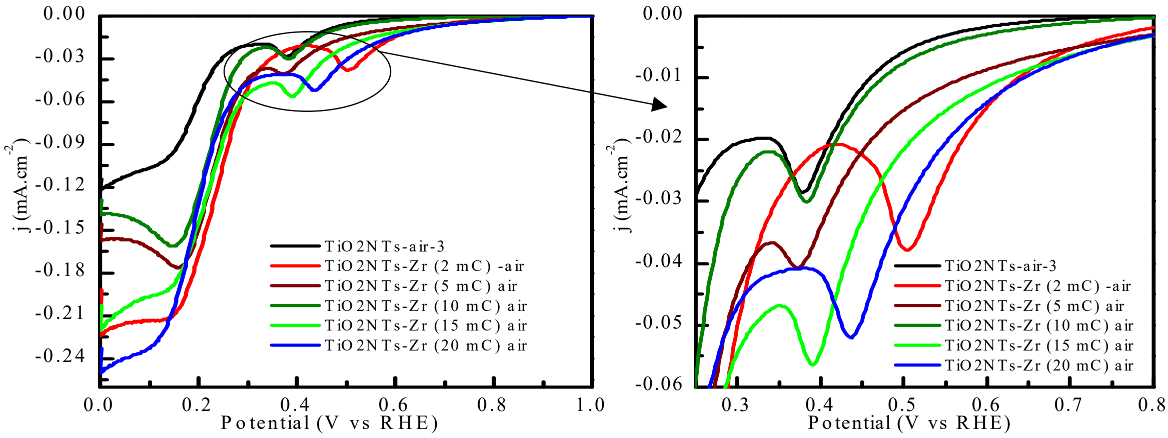

Figure S1. LSVs for a TNTs electrode with different ZrO_2 loading (2 to 20 mC) at scan rate 10 mVs^{-1} immersed in 1.0 M NaOH and annealed under air
